# Analysis of Cyclin-Dependent Kinase 1 as an Independent Prognostic Factor for Gastric Cancer Based on Statistical Methods

**DOI:** 10.3389/fcell.2020.620164

**Published:** 2020-12-07

**Authors:** Xu Zhang, Hua Ma, Quan Zou, Jin Wu

**Affiliations:** ^1^School of Mathematics and Statistics, Southwest University, Chongqing, China; ^2^Institute of Fundamental and Frontier Sciences, University of Electronic Science and Technology of China, Chengdu, China; ^3^Hainan Key Laboratory for Computational Science and Application, Hainan Normal University, Haikou, China; ^4^School of Management, Shenzhen Polytechnic, Shenzhen, China

**Keywords:** CDK1, prognosis, biomarker, gastric cancer, bioinformatics

## Abstract

**Objective:**

The aim of this study was to investigate the expression of cyclin-dependent kinase 1 (CDK1) in gastric cancer (GC), evaluate its relationship with the clinicopathological features and prognosis of GC, and analyze the advantage of CDK1 as a potential independent prognostic factor for GC.

**Methods:**

The Cancer Genome Atlas (TCGA) data and corresponding clinical features of GC were collected. First, the aim gene was selected by combining five topological analysis methods, where the gene expression in paracancerous and GC tissues was analyzed by Limma package and Wilcox test. Second, the correlation between gene expression and clinical features was analyzed by logistic regression. Finally, the survival analysis was carried out by using the Kaplan–Meier. The gene prognostic value was evaluated by univariate and multivariate Cox analyses, and the gene potential biological function was explored by gene set enrichment analysis (GSEA).

**Results:**

CDK1 was selected as one of the most important genes associated with GC. The expression level of CDK1 in GC tissues was significantly higher than that in paracancerous tissues, which was significantly correlated with pathological stage and grade. The survival rate of the CDK1 high expression group was significantly lower than that of the low expression group. CDK1 expression was significantly correlated with overall survival (OS). CDK1 expression was mainly involved in prostate cancer, small cell lung cancer, and GC and was enriched in the WNT signaling pathway and T cell receptor signaling pathway.

**Conclusion:**

CDK1 may serve as an independent prognostic factor for GC. It is also expected to be a new target for molecular targeted therapy of GC.

## Background

Gastric cancer (GC) is a very common malignant tumor, and its prognosis is relatively poor. In 2015, it was found that the incidence rate of GC was second in all cancers (Chen et al., 2015; Lin et al., 2019; Mottini et al., 2019). Due to the hidden nature of the disease, the early symptoms are not obvious. Most of patients were in the advanced stage at the time of treatment, and the 5-year overall survival (OS) rate was only 28.3% (Siegel and Naishadham, 2012). GC was prone to lymph node metastasis and had strong invasive ability (Cutsem et al., 2015). At present, many studies have focused on identifying new biomarkers for early diagnosis and prognosis prediction of GC (Verma and Sharma, 2018; Ji et al., 2019; Li et al., 2019; Ma et al., 2019; Zheng et al., 2019). However, no widely accepted biomarkers have been found. Therefore, it is very important to identify effective biomarkers for the diagnosis and prognosis of GC (Zou et al., 2016; Zeng et al., 2017, 2018; Zhang et al., 2017; Tang et al., 2018; Xu et al., 2018, 2019).

Cyclin-dependent kinase 1 (CDK1) gene is a cyclin kinase, which can lead to malignant cell proliferation after activation (Ling et al., 2013). It was shown that CDK1 had a positive regulatory effect on the cell cycle of GC, and that its abnormal activation was involved in the malignant transformation of GC. Fu et al. focused on the relationship between the expression of CDC25A and CDK1 and lymph cancer patients. They found that CDC25A and CDK1 were highly expressed in GC tissues with lymph nodes and lowly expressed in GC without lymph nodes. Wang et al. revealed that the high expression of Cyclin B2 and CDK1 in GC patients may indicate that the biological ability of tumor invasion was strong and was related to the low OS rate of patients. Gao et al. (2014) investigated that the downregulation of CDK1 and cyclin B1 expression contributed to oridonin-induced cell cycle arrest at the G2/M phase and growth inhibition in SGC-7901 GC cells. However, so far, no study has proposed and tested CDK1 as an independent prognostic factor for GC, and the molecular mechanism of CDK1 in GC is still unclear. Therefore, the aim of this study is to explore the advantages of CDK1 as an independent prognostic factor for GC from the point of view of statistics and bioinformatics.

In this paper, CDK1 was selected by the intersection of five topological analysis methods in CytoHubba plug-in. The expression of CDK1 in paracancerous tissues and GC tissues was compared, and its correlation with clinical features was studied by non-parametric test. Then, we investigated the prognostic value of CDK1 from analyzing the correlation of its expression with OS by univariate and multivariate Cox analyses and explored the potential biological function of CDK1 using the gene set enrichment analysis (GSEA).

## Materials and Methods

### Dataset

We collected the set of gene expression profiles of GC from the Gene Expression Omnibus database^1^. This dataset included 10 GC samples and 10 normal samples. The platform was GPL570 (Affymetrix Human Genome U133 Plus 2.0), and The Cancer Genome Atlas (TCGA) data with corresponding clinical features of GC were downloaded from TCGA database^2^ that contained 375 tumor tissue samples and 32 paracancerous tissue samples. Gene symbol annotation information was used to match the corresponding probe, gene expression (Workflow Type: HTSeq-FPKM), and related clinical information (Data Type: Clinical Supplement) data (Wang et al., 2016). TCGA database is publicly available and is an open access platform.

### Gene Selection

First, the Limma method was used to identify differentially expressed genes (DEGs) between GC tissues and paracancerous tissues. Second, a protein–protein interaction (PPI) network of DEGs was constructed based on the STRING V11 database, and the clustering module of the hub genes was obtained using the Molecular Complex Detection (MCODE) method in Cytoscape software (Shannon et al., 2003). Finally, five topological analysis methods in the CytoHubba plug-in (Chin et al., 2014), including Closeness, Maximal Clique Centrality (MCC), Maximum Neighborhood Component (MNC), Degree, and Edge Percolated Component (EPC), were used to narrow down the gene shortlist further, and the top-ranked genes were compared and selected for downstream analysis.

### Gene Expression in Paracancerous Tissues and GC Tissues

To compare the gene expression levels in GC and paracancerous tissues, first, the single-gene expression data were extracted using the Limma package, and the data were divided into the high and low expression groups using the median value (samples with the gene expression greater/less than the median value were considered as the high/low expression group). Second, the *p*-value of Wilcox test was calculated where *p* < 0.05 indicated that the gene expression was significantly different between GC tissues and paracancerous tissues. Beeswarm package was used to draw scatter plots for differential expression analysis. Finally, in order to prove the conclusion further, the Perl script was used to obtain the paired samples of paracancerous tissues and GC tissues, and the differential expression analysis plot of the paired samples was drawn.

### Correlation Analysis Between Gene Expression and Clinical Features

A large amount of clinicopathological information including gender, age, tumor-node-metastasis (TNM) staging, grade, and pathological stage were collected from TCGA dataset. Logistic regression was used to test the correlation between gene expression and the clinical features where a *p*-value < 0.05 was considered statistically significant.

### Survival Analysis

In the survival analysis, all the paracancerous tissue samples were removed, and only the GC tissue samples were retained. Based on the high and low expression groups, Kaplan–Meier was used to draw a survival analysis curve of the selected gene where a *p*-value of 0.05 was used as the statistical threshold.

### Univariate and Multivariate Cox Analyses

Cox proportional hazard models of univariate and multivariate were used to calculate 95% confidence interval (CI) and hazard ratio (HR) where survival package was used for statistical analysis. Univariate Cox analysis model was used to compare the relationship between clinical features and survival rates. Multivariate Cox analysis model was used to evaluate how the gene expression and the clinical factors (gender, age, grade, and stage) affect OS. *p* < 0.05 was set as the threshold, and the forest boxplot was drawn using the survminer package.

### Gene Enrichment Analysis

The data obtained from TCGA were divided into the high and low expression groups according to the expression of target gene for multi-factor GSEA (Subramanian, 2005). GSEA was carried out to explore the gene biological functions. The enrichment results that satisfied two conditions of FDR < 0.05 and *p* < 0.05 were considered statistically significant.

## Results

### Selection of CDK1 Gene

**FIGURE 1:**
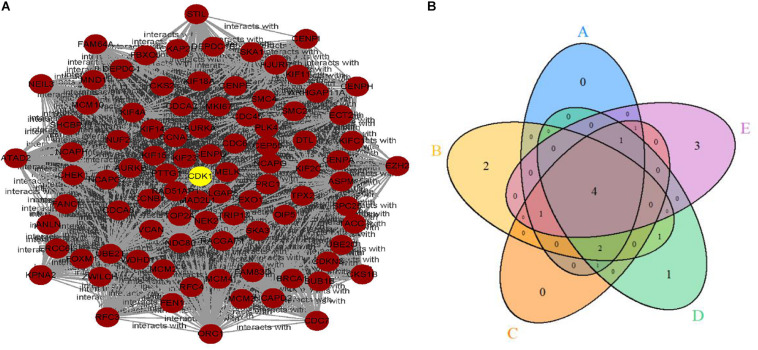
**(A)** The clustering module of the hub genes with the highest score generated by MCODE. **(B)** The intersection of the top 10 selected genes of five topological methods.

Limma identified 1,599 DEGs in the dataset GSE79973 where 1,269 genes were upregulated and 330 genes were downregulated. These DEGs were imported into the STRING V11 database to obtain a TSV file of protein interactions. After the hub genes were calculated by CytoHubba plug-in, one cluster module of hub genes with the highest scoring was obtained that contained 92 nodes/genes and 3,628 edges (Figure 1A). The 92 hub genes were calculated by five topological analysis methods, and the top 10 ranked genes for each method were selected (Table 1), among which CDK1, VCAN, CCNB1, and AURKB were found in the intersection of the results of five methods (Figure 1B). All the four hub genes were upregulated. Besides, CDK1 was found to be ranked first by two topological methods (MNC and EPC) (Table 1). Therefore, in the downstream analysis, we focused on the expression and prognostic value of CDK1 in GC.

**TABLE 1:** Top 10 genes and their scores selected by Degree, MCC, EPC, MNC, and Closeness methods.

Rank	Degree	Score	MCC	Score	EPC	Score	MNC	Score	Closeness	Score
1	VCAN	174	CCNB1	2.08E + 74	CDK1	193.496	CDK1	173	FN1	622.45
2	CDK1	173	AURKB	2.08E + 74	CCNB1	191.605	VCAN	172	VCAN	607.0666667
3	FN1	162	CDK1	2.08E + 74	AURKB	191.534	FN1	161	CDK1	590.9833333
4	AURKA	157	BUB1B	2.08E + 74	BUB1B	191.227	AURKA	155	AURKA	590.05
5	CCNB1	148	NCAPG	2.08E + 74	AURKA	190.784	CCNB1	147	CCNB1	574.0833333
6	CCNA2	143	CCNA2	2.08E + 74	TOP2A	190.703	CCNA2	143	NOTCH1	571.1833333
7	AURKB	141	MAD2L1	2.08E + 74	CDC6	190.141	AURKB	141	AURKB	570.1166667
8	MAD2L1	140	KIF11	2.08E + 74	VCAN	189.837	MAD2L1	139	CCNA2	562.4833333
9	BUB1B	138	VCAN	2.08E + 74	CDCA8	189.695	BUB1B	137	MKI67	561.9666667
10	TOP2A	134	CDCA8	2.08E + 74	MAD2L1	189.499	TOP2A	134	BRCA1	561.2166667

### Expression of CDK1 in Paracancerous Tissues and GC Tissues

**FIGURE 2:**
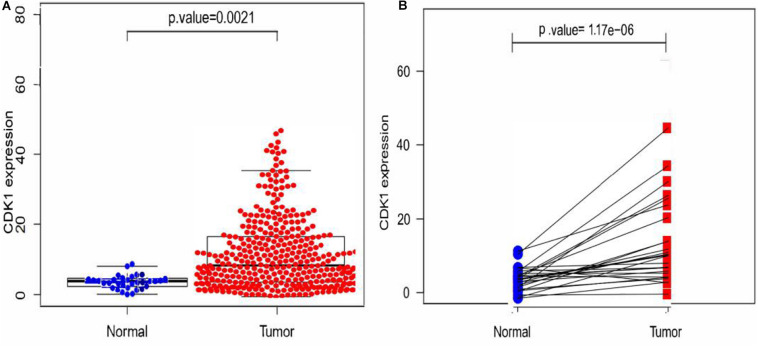
Expression analysis of CDK1. **(A)** The scatter plot of differential expression analysis of CDK1 in two types of tissues. **(B)** The differential expression analysis map of paired samples. Blue dots represent the paracancerous tissues, and red dots represent the GC tissues.

Since the median value of the expression level of CDK1 in GC tissues was significantly higher than that in paracancerous tissues, CDK1 was considered to be highly expressed in GC tissues (Figure 2A). This conclusion could also be drawn from the differential expression analysis of paired tissue samples (Figure 2B), where the lines connected the paracancerous tissue and GC tissue of the same patient. Most lines had an upward trend indicating that the expression level of CDK1 was highly expressed in GC tissues.

### Correlation Between CDK1 Expression Level and Clinicopathological Features

According to clinical and pathological information from TCGA, logistic regression analysis showed that CDK1 expression level was significantly correlated with stage (*p*-value < 0.05 for III vs I and IV vs I) and grade (*p*-value < 0.05 for III vs I), but not significantly correlated with age and gender (*p* > 0.05, Table 2).

**TABLE 2:** Correlation between CDK1 expression and clinicopathological features using logistic regression.

Clinical characteristic	Total (N)	Odds ratio in CDK1 expression	*p*-Value
Stage (II vs I)	352	2.22 (1.35–3.62)	0.0501
Stage (III vs I)	352	2.14 (2.13–3.96)	0.0491
Stage (IV vs I)	352	2.01 (1.95–2.24)	0.0243
Gender	298	2.28 (2.30–3.33)	0.0941
Age	343	1.98 (1.01–2.31)	0.0501
Grade (II vs I)	263	2.39 (2.52–4.37)	0.0691
Grade (III vs I)	263	2.32 (2.14–2.32)	0.0325
Grade (IV vs I)	263	1.81 (2.12–4.02)	0.0524

### Survival Analysis of CDK1

**FIGURE 3:**
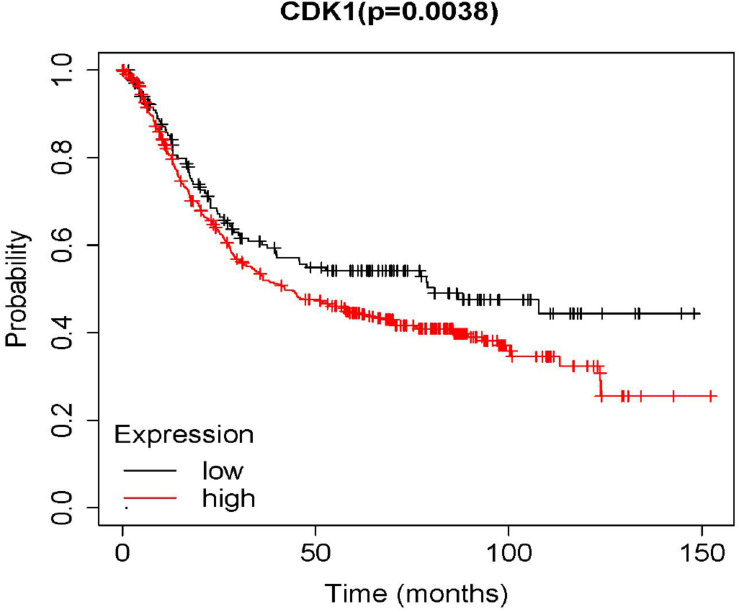
The overall survival rate of the high and low expression groups of CDK1.

In the result of survival analysis of CDK1 (Figure 3), *p* < 0.05 indicated that the survival rates of the high and low expression groups were significantly different. The red line represented the high expression group, and the black line represented the low expression group. It was seen from the result in the figure that the survival rate of the high expression group was significantly lower than that of the low expression group.

### Univariate and Multivariate Cox Analyses

**FIGURE 4:**
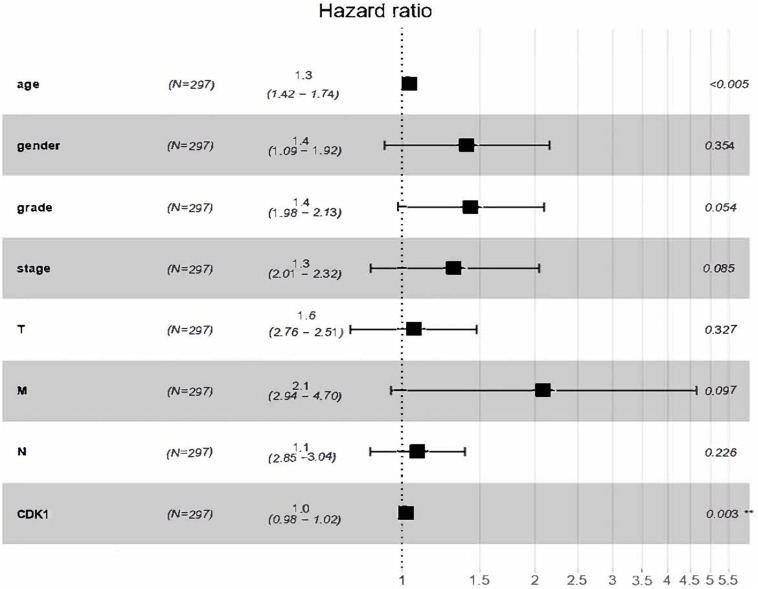
Forest boxplot for multivariate Cox analysis of the correlation between CDK1 expression and OS as well as other clinical features.

The analysis results of the correlation between CDK1 expression and OS as well as other clinical features investigated by Cox models were shown in Table 3. In univariate survival analysis, some factors including age (HR = 1.269, *p*-value = 0.0056), pathological stage (HR = 1.3350, *p*-value = 0.0054), T (HR = 1.0975, *p*-value = 0.0317), N (HR = 1.6707, *p*-value = 0.0087), M (HR = 2.4306, *p*-value = 0.0245), and CDK1 expression (HR = 1.2182, *p*-value = 0.0023) were revealed to be significantly correlated with OS. And the multivariate Cox analysis, described by the forest boxplot (Figure 4), also suggested that age and CDK1 expression were significantly correlated with OS (*p*-value < 0.05). Therefore, CDK1 may serve as an independent prognostic factor for GC.

**TABLE 3:** Univariate and multivariate Cox analyses of the correlation between CDK1 expression and OS as well as other clinical features.

	Univariate Cox				Multivariate Cox			
Variables	HR	HR.95L	HR.95H	*p*-Value	HR	HR.95L	HR.95H	*p*-Value
Age	1.2696	1.7830	2.0464	0.0056	1.3049	1.4190	1.7411	0.0047
Gender	1.4838	1.9797	2.2471	0.0627	1.4024	1.0911	1.9156	0.3541
Grade	1.0678	0.9465	1.9763	0.0953	1.3691	1.9786	2.1309	0.0543
Stage	1.3350	1.2213	1.9310	0.0054	1.3163	2.0143	2.3224	0.0854
T	1.0975	1.3345	1.6208	0.0317	1.6046	2.7625	2.5142	0.3270
M	2.4306	1.9617	3.8376	0.0245	2.0915	2.9428	4.7041	0.0974
N	1.6707	1.0695	1.5011	0.0087	1.0833	2.8459	3.0387	0.2257
CDK1	1.2182	1.0707	1.3641	0.0023	1.0100	0.9756	1.0243	0.0031

*TNM, tumor, node, and metastasis classification.*

### Multi-Factor GSEA Enrichment Analysis

**FIGURE 5:**
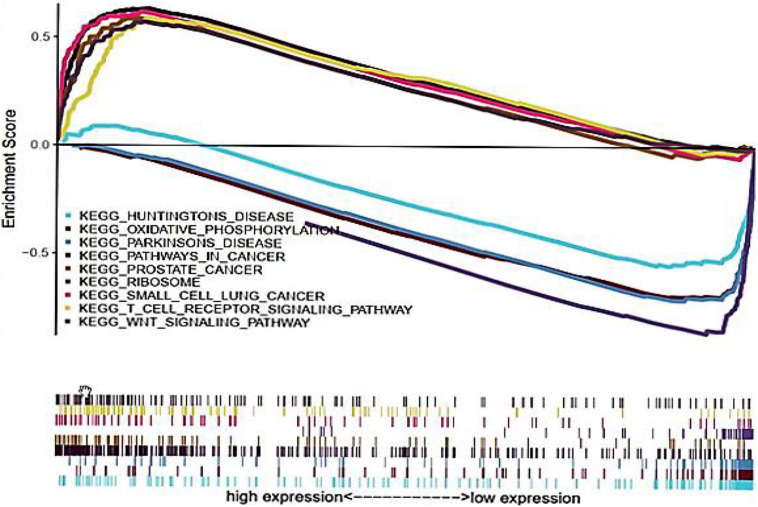
KEGG pathway shows five positive and four negative correlated groups.

Gene set enrichment analysis was performed to explore the potential biological functions of CDK1, and it was used to analyze the high and low expression groups of CDK1 gene. Figure 5 shows the result of Kyoto Encyclopedia of Genes and Genomes (KEGG) pathway analysis with top five pathways positively related to CDK1 expression and top four negatively related pathways. These results implied that CDK1 expression was involved in prostate cancer, small cell lung cancer, and GC and was significantly correlated with the WNT signaling pathway and T cell receptor signaling pathway (Table 4).

**TABLE 4:** Enrichment parameters of CDK1 analyzed by GSEA.

Gene set name	ES	NES	*p*-Value	FDR q-value
KEGG_PATHWAYS_IN_CANCER	0.6324	2.3544	0.0000	0.0000
KEGG_WNT_SIGNALING_PATHWAYS	0.5718	2.1285	0.0000	0.0012
KEGG_SMALL_CELL_LUNG_CANCER	0.6173	2.0532	0.0000	0.0029
KEGG_PROSTATE_CANCER	0.5882	2.0019	0.0000	0.0000
KEGG_T_CELL _RECEPTOR_SIGNALING_PATHWAYS	0.5907	1.8971	0.0119	0.0083
KEGG_PARKINSONS_DISEASE	−0.7210	−2.0901	0.0020	0.0261
KEGG_OXIDATIVE_PHOSPHORYLATION	−0.7011	−2.0433	0.0020	0.0260
KEGG_RIBOSOME	−0.8701	−1.9910	0.0080	0.0350
KEGG_HUNTINGTONS_DISEASE	−0.5491	−1.9785	0.0040	0.0263

## Discussion

Gastric cancer is one of the common digestive malignancies that seriously threaten human health. However, the factors affecting the occurrence, development, and prognosis of GC are still unclear. Therefore, it is of great significance to explore the genes related to the prognosis of GC from the gene level and molecule level for the treatment and prognosis assessment of GC.

The Cancer Genome Atlas database contains abundant types of tumor data and complete clinical information, which provides an important resource for the study of GC. In this study, the significant role of CDK1 in the treatment and prognosis of GC was discussed by comprehensive statistical methods. First, CDK1 was selected as the aim gene by combing five topological methods. The results of Limma package and Wilcox test showed that CDK1 was highly expressed in GC samples. Second, logistic regression was used to analyze the correlation between CDK1 expression and the corresponding clinical features. It was concluded that CDK1 expression was significantly correlated with pathological stage and grade, but not with age or gender. Survival analysis using Kaplan–Meier showed that the survival rate of the CDK1 high expression group was significantly lower than that of the low expression group. And the prognostic value of CDK1 was analyzed by univariate and multivariate Cox proportional hazard models. The results showed that CDK1 may be an independent prognostic factor for GC. Finally, GSEA revealed that CDK1 expression was involved in prostate cancer, small cell lung cancer, and GC and was significantly correlated with the WNT signaling pathway and T cell receptor signaling pathway.

There were several literatures using the same dataset in this study (Chen et al., 2020; Chi et al., 2020; Li Z. et al., 2020; Tian et al., 2020; Wang F. et al., 2020; Wu et al., 2020). However, most of them selected multiple genes for GC by some routine methods and did not concentrate on one gene/biomarker. And CDK1 was not identified as a key gene associated with GC in those studies. In view of the important role of CDK1 in the prognosis evaluation of GC, it may become a new target for precise treatment of GC, which is worthy of further study. Some references have shown that the abnormal expression of CDK1 was associated with poor prognosis of some other cancers including colorectal cancer, lung cancer, and pleural mesothelioma. Zhang et al. found that the loss of cytoplasmic CDK1 predicted low survival rate of human lung cancer and induced chemotherapeutic resistance (Chunyu et al., 2011). Linton et al. (2014) revealed through an RNAi-based screen that PLK1, CDK1, and NDC80 may be the potential therapeutic targets in malignant pleural mesothelioma. Sung et al. (2014) showed that high nuclear/cytoplasmic ratio of CDK1 expression predicted poor prognosis in colorectal cancer. Nishida et al. (2015) found that cyclin-dependent kinase activity was related to the prognosis of gastrointestinal tumors. These studies provided theoretical support for CDK1 as a therapeutic target and a new prognostic factor for GC. However, the expression and role of CDK1 in GC are still not fully understood. Therefore, this study evaluated the correlation between CDK1 and prognosis of GC as well as other clinicopathological features from the point of view of statistics and bioinformatics (Wang et al., 2013; Wei et al., 2017a,b, 2018; Su et al., 2018; Zhang et al., 2018; Ding et al., 2019; Shen et al., 2019; Fan et al., 2020; Li H. Y. et al., 2020; Li J. et al., 2020; Tan et al., 2020; Wang H. et al., 2020; Wang Z. et al., 2020) and provided important clues for further exploring the biological function and molecular mechanism of CDK1. In future work, if condition permits, we hope to conduct some experiments to verify the important role of CDK1 in GC from biological point of view.

## Data Availability Statement

The original contributions presented in the study are included in the article/supplementary material. Further inquiries can be directed to the corresponding author/s.

## Ethics Statement

Ethical review and approval was not required for the study on human participants in accordance with the local legislation and institutional requirements. Written informed consent for participation was not required for this study in accordance with the national legislation and the institutional requirements.

## Author Contributions

XZ contributed to data analysis, methodology, and article writing. HM contributed to investigation, figures construction, and validation who contributed equally with XZ. QZ contributed to methodology and validation. JW contributed to supervision and writing of the article. All authors contributed to the article and approved the submitted version.

## Conflict of Interest

The authors declare that the research was conducted in the absence of any commercial or financial relationships that could be construed as a potential conflict of interest.

## Footnotes

1 https://www.ncbi.nlm.nih.gov/geo/query/acc.cgi?acc=GSE79973

2 https://tcgadata.nci.nih.gov/tcga/
